# Impact of China’s zero mark-up drug policy on drug cost of NCDs’ outpatients: an interrupted time series analysis

**DOI:** 10.1186/s12913-021-06414-3

**Published:** 2021-04-29

**Authors:** Jielin Du, Jiajia Xie, Yan Qian, Mingyue Wu, Wenjing Huang, Jin Yin, Xin Peng, Dan Deng

**Affiliations:** 1 School of Public Health and Management, Chongqing Medical University, Chongqing, China; 2 Sichuan Tianfu New Area Center for Disease Control and Prevention, Chengdu, Sichuan China; 3 Department of Pharmacy, The Second Affiliated Hospital of Chongqing Medical University, Chongqing, China

**Keywords:** Zero mark-up drug policy, Interrupted time series, Chronic noncommunicable diseases, Policy evaluation, China

## Abstract

**Background:**

China proposed the Zero Markup Drug Policy (ZMDP), which popularized in tertiary hospitals across the country in 2017, to control drug expenditures’ rapid growth further and reduce the public’s medical burden. This study aims to evaluate the impact of ZMDP on the drug cost of chronic disease outpatients in the tertiary hospital in Chongqing.

**Methods:**

We collected and described the drug-cost data for outpatients with chronic diseases in a Chongqing’s tertiary hospital from 2015 to 2019. The instantaneous and long-term changes of the outpatient volume and average drug cost after the ZMDP were evaluated using interrupted time series (ITS). We also analyzed the policy’s impact under the stratification of gender, age, and basic medical insurance types.

**Results:**

A total of 350,848 outpatients were collected from January 2015 to February 2019. After the ZMDP, the outpatient volume for diabetes, hypertension, and coronary heart disease (CHD) all showed a downward trend, with a decrease of 53.04 (*P* = 0.012), 142.19 (*P* < 0.01) and 12.16 (*P* < 0.001) per month. Simultaneously, the average drug cost decreased by 4.44 yuan (*P* = 0.029), 5.87 yuan (*P* < 0.001) and 10.23 yuan (*P* = 0.036) per month, respectively. By gender, the average drug cost of diabetes in males had the most considerable instantaneous change, reducing by 51.21 yuan (*P* = 0.017); the decline of CHD in women is the most obvious, with an average monthly decrease of 12.51 yuan (*P* < 0.001). By age, the instantaneous change of CHD was the greatest for those older than 65 years old, with a decrease of 102.61 yuan (*P* = 0.030). CHD in 46–65 years old showed the most significant reduction, with an average monthly decline of 11.70 yuan (*P* < 0.01). BMIUE’s hypertension had the most considerable instantaneous change, which decreased 59.63 yuan (*P* = 0.010). BMIUE’s CHD showed the most apparent downward trend, with an average monthly decrease of 10.02 yuan (*P* = 0.010).

**Conclusion:**

The ITS analysis is an effective method of health policy evaluation. The implementation of the ZMDP can reduce the drug cost for chronic disease outpatients in the tertiary hospital and their economic burden. Follow-up policies still require targeted price adjustments in the health service system to adjust the drug cost-effectively.

## Background

Since 1954, China proposed a drug mark-up policy that allowed medical and health institutions at and above the county level to sell drugs at a rate that did not exceed 15% of the drug’s actual per-unit purchase price [1]. This policy aims to remedy insufficient compensation in public hospitals and maintain their survival and development. The drug mark-up policy has played a positive role in improving hospital operations during Chinese public hospitals’ development process. However, the pharmaceutical and healthcare fields’ development for decades has found that the drug mark-up policy has led to profit-seeking behavior in hospitals to a certain extent [2]. It has also led to a series of problems, including an increasing medication burden for patients, a high total cost for medical treatment, and a gradual increase in the tension between doctors and patients [3]. Drug expenditure per capita has grown rapidly, and drug revenue has become the primary funding source for public hospitals. In 2008, drug expenditures accounted for a staggering 42.67% of total health care expenditures [4]. Reducing the cost of medicine is one of the keys to solving the problem of increasing medical costs caused by the excessive growth of drug costs [5].

In 2009, China launched its essential medicine plan and started a new round of medical and healthcare reforms [6]. The Zero Mark-up Drug Policy (ZMDP), as a necessary part of the essential drug plan, has been implemented in a step-wise manner beginning with primary medical and health institutions. The plan’s core goal is to compensate for decoupling prescription writing and drug sales, thereby reducing the pursuit of intermediate drug benefits and ultimately reducing the public’s medical burden [7]. In 2017, the PRC’s State Council required nationwide public hospitals to abolish drug mark-up fees (except Chinese herbal medicines) before September 30. To date, all public hospitals across the country have eliminated drug mark-ups. Since the implementation of ZMDP, research on its effectiveness evaluation has become a hot topic. Some studies showed that ZMDP is an effective intervention that can suppress the increase in cost [2, 6]. However, some argue that the impact of ZMDP on drug-related spending and use in public hospitals is not significant [8].

The ZMDP plays an important role in reducing the drug cost of chronic noncommunicable diseases (chronic diseases, NCDs), such as diabetes, hypertension, coronary heart disease (CHD) and cancer [9]. NCDs seriously threaten the physical and mental health of people around the world because of their high morbidity, high mortality, long-term nature, complex etiology and long-term medication [10]. It is a common and significant public health problem worldwide. NCDs ranked third on the WHO’s list of global health threats in 2019 [11]. Similarly, the proportion of deaths due to NCDs in China may be as high as 86.6%, which is much higher than the global level, and the number of patients with NCDs is increasing [12]. NCDs’ clinical characteristics, such as difficulty estimating their progress and an inability to cure the majority of them, mean that most patients need long-term or even lifelong medications [9]. Therefore, NCD drug expenses account for a large proportion of medical insurance expenses. Medical expenses for hypertension, diabetes, and cardiovascular and cerebrovascular diseases alone accounted for 12.5% of the total national health expenditure, among which self-administered drugs for hypertension and diabetes accounted for more than 50% of medical expenses [13]. Therefore, for chronic disease patients, drug costs account for the vast majority of their medical costs. The long-term, high medical expenses have plunged families into poverty.

The drug cost for NCDs accounts for a large proportion of all medical costs, and this situation is expected to continue for a long time. However, current studies on NCDs’ medical expenses both at home and abroad still focus on the factors influencing NCDs’ hospitalization expenses, forecasts of hospitalization expenses, or the composition of costs [14, 15]. At the same time, there are few studies on outpatient expenses [16, 17]. Outpatient can reflect the overall scale, service quality and medical-technology level of the hospital. They are the first step in developing diagnoses and treatments and are also an effective opportunity for the hospital to conduct external business [18, 19]. According to national statistics, drug costs in Zhejiang, Chongqing, and Jiangsu, among others, accounted for 51.75 to 74.37% of total outpatient expenses, and drug costs in all cities exceeded 50% of outpatient expenses in recent years in China [20–22].

**Fig. 1:**
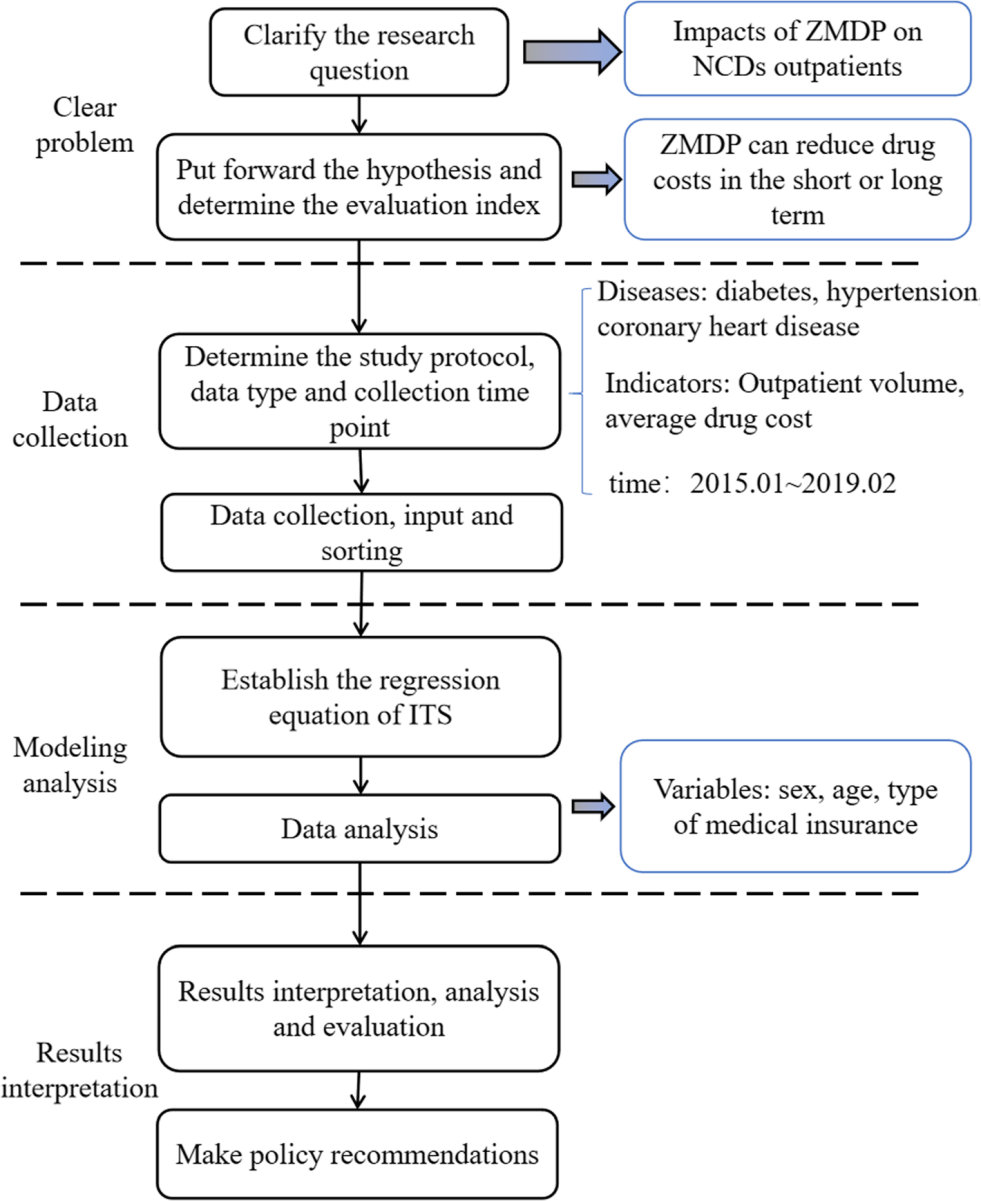
The flowchart of ITS analysis of drug costs on NCDs outpatients

The ZMDP started in primary medical and health institutions, moved to secondary hospitals, and then to all county-level hospitals across the country [23]. The tertiary hospital is the final implementer of ZMDP, patients with chronic diseases are the greatest beneficiaries of the ZMDP. As an essential strategic fulcrum for the western Chinese region, Chongqing lacks relevant research. Therefore, it is indispensable to analyze the effect of NCDs’ outpatient drug cost after the ZMDP in Chongqing’s tertiary hospital. Given that the patient’s condition, such as gender, age, etc., is considered, this study intends to evaluate the impact of ZMDP on the average drug cost of multiple chronic disease outpatients based on interrupted time series analysis. Specifically, collect the drug cost of the three most common chronic diseases (diabetes, hypertension, and CHD) in the outpatient of a tertiary hospital in Chongqing. Five models were constructed to analyze the outpatient volume changes, total average drug costs, average drug costs by sex, average drug costs by age, and average drug costs by medical insurance type for the three diseases after the policy. Finally, it analyzes and compares the differences and puts forward targeted suggestions, aiming to reference decision-makers from the relevant industries and departments to refer to as they continue to deepen the medical and health system’s reform. The flowchart is shown in Fig. 1.

## Methods

### Setting and study population

Chongqing is a megacity with a population of more than 30 million. It is also the only municipality directly under the Central Government’s governance in the Midwest and is one of China’s major central cities. This study explores the impact of the ZMDP on the average drug cost for NCDs outpatients in tertiary hospitals in Chongqing. To avoid the impact of comorbid diseases on drug cost, all diagnoses containing comorbidities were excluded from this study. The top 3 chronic diseases among outpatients were selected for the research focus, namely, diabetes (ICD-10 coded as E10-E14), hypertension (ICD-10 coded as I10), and coronary atherosclerotic heart disease (abbreviated as coronary heart disease, ICD-10 coded as I25).

### Outcome variables and data sources

The outcome measure is defined as the average drug cost1^1^ per month for NCDs’ outpatients analyzed overall. The data are stratified according to gender, age and type of basic medical insurance, too. The data come from the hospital information system. From January 2015 to February 2019, 50 monthly observations were collected, sufficient for interrupted time series analysis. This study used the period when the ZMDP was first implemented in Chongqing’s tertiary hospital in September 2017 as the intervention point. Interrupted time series analysis is used to estimate both the immediate (change in outcome levels) and long-term (change in outcome trends) effects of the policy.

### Statistical analysis

Interrupted time series (ITS) analysis is a quasi-experimental research design proposed by Box and Tiao in 1975 to evaluate the effects of certain interventions retrospectively [24]. Its purpose is to determine whether the changes observed in the outcomes can be explained by long-term trends or are attributable to the intervention measures themselves. This is done by evaluating changes in the outcomes’ levels and trends before and after the intervention measures [25]. The segmented regression time series model is one of the most commonly used ITS methods. It constructs multiple regression equations to perform regression analyses on the periods before and after the intervention measures, which requires fewer periods and relatively simple model building [26–28]. The segmented regression model based on the intervention period being in September 2017 is as follows:

$$ {Y}_t={\beta}_0+{\beta}_1\times {X}_{time}+{\beta}_2\times {X}_{ZMDP}+{\beta}_3\times {X}_{posttime}+{e}_t $$

Where *Y*_*t*_ is the outcome variable for the study at time t; *X*_*time*_ is the continuous-time variable, which represents the unit of time (such as months, years, etc.) used in the study, *X*_*time*_ = 1, 2, 3…*n* . This study uses months as the unit, and the values of the observations are from 1 to 50; *X*_*ZMDP*_ is a binary dummy variable, where the months before the intervention are represented by 0 (i.e., *X*_*ZMDP*_ = 0), and those after are represented by 1 (i.e., *X*_*ZMDP*_ = 1), with the change taking effect in the 33rd month of the series; *X*_*posttime*_ is used in the time series analysis to count the months after the intervention. Months before the intervention are represented by 0 (i.e., *X*_*posttime*_ =0), and the months after the intervention are *X*_*posttime*_ = 1, 2, 3…*n*, which ranges from 1 to 18 in this study.

*β*_0_ represents a constant, and t = 0 reflects the baseline level; *β*_1_ represents the estimated time trend before the intervention, that is, the slope of *X*_*time*_ before the intervention; *β*_2_ represents the instantaneous level change due to the intervention; and *β*_3_ reflects the time trend change after the intervention is implemented, that is, the amount by which the slope changes. *e*_*t*_ is the random error value. The data analysis was performed using the Statistical Analysis System, version 9.4 (SAS, North Carolina State University, USA). The level of significance was set *P* < 0.05.

## Results

From January 2015 to February 2019, the outpatient volume for the top three chronic diseases in a tertiary hospital in Chongqing was 350,848, including 197,227 diabetes, 130,993 hypertension and 22,628 CHD. Separately, among all 197,227 diabetic outpatients, 96,072 were women and 101,155 were men. There were 101,838 diabetes aged 46–65 years, accounting for 51.63% of the total. The majority of medical payments are through BMIUE (182,432 person-visits in total). Second, among all 130,993 hypertension outpatients, 69,600 were women and 61,393 were men. Patients were mainly 46 years old or older. The cumulative BMIUE patients were 122,273, and the BMIRC patients were the lowest, only 232. Finally, among all 22,628 CHD outpatients, 8836 were women and 13,792 were men. More than half of the patients were over 65 years old, with 12,676 patients. Like diabetes and hypertension, most patients use BMIUE for payment, accounting for 89.87% of the total.

The total average drug cost of outpatients for treating chronic diseases was 688.65 yuan. Diabetes, hypertension and CHD were 736.56 yuan, 601.00 yuan and 778.36 yuan, respectively. Among diabetic outpatients, the average drug cost was 736.56 yuan, 720.06 yuan for males, and 753.94 yuan for females. Among hypertension outpatients, the average drug cost was 601.00 yuan, 607.45 yuan for males, and 595.31 yuan for females. Among CHD outpatients, the average drug cost was 778.36 yuan, 805.85 yuan for males, and 735.46 yuan for females. Diabetic, hypertension and CHD all have the highest average drug costs for those over 65 years old, which are 822.62 yuan, 690.01 yuan and 796.56 yuan respectively. Similarly, the average drug costs of BMIRC are the highest for the three diseases, which are 1029.62 yuan, 675.23 yuan and 977.25 yuan respectively. As shown in Table 1, the three diseases’ outpatient volume and average drug costs are quite different. Therefore, this study analyzes the three diseases separately better to compare the impact of the implementation of the ZMDP.

**Table 1:** Outpatient volume and the average drug cost for three chronic diseases from January 2015 to February 2019

Indicator		Outpatient volume (person-month)				Average drug cost (RMB)			
		Total	Diabetes	Hypertension	Coronary heart disease	Total	Diabetes	Hypertension	Coronary heart disease
Sex	Female	174,508	96,072	69,600	8836	689.74	753.94	595.31	735.46
	Male	176,340	101,155	61,393	13,792	687.56	720.06	607.45	805.85
Age	< 46	25,024	15,380	9040	604	468.51	535.50	346.92	582.68
	46–65	169,601	101,838	58,415	9348	649.35	699.32	543.50	766.34
	> 65	156,223	80,009	63,538	12,676	766.57	822.62	690.01	796.56
Basic medical insurance type	BMIUE	325,040	182,432	122,273	20,335	712.42	763.34	621.26	803.72
	BMIUR	12,790	8548	3245	997	376.81	390.80	291.68	533.91
	BMIRC	806	417	232	157	917.41	1029.62	675.23	977.25
	Self-insured	12,212	5830	5243	1139	367.44	384.82	316.65	512.26
Year	2015	89,795	46,237	38,335	5223	704.56	772.03	609.88	802.17
	2016	89,077	46,607	36,598	5872	711.89	775.03	619.63	785.73
	2017	91,310	49,679	35,675	5956	684.35	731.93	597.58	807.23
	2018	71,809	47,951	18,927	4931	656.25	684.80	564.39	731.30
	2019 (Jan-Feb)	8857	6753	1458	646	600.43	629.96	458.56	611.99
ZMDP implemented	NO	242,006	126,979	99,728	15,299	707.74	769.24	614.99	801.88
	YES	108,842	70,248	31,265	7329	646.20	677.50	556.38	729.28
Total		350,848	197,227	130,993	22,628	688.65	736.56	601.00	778.36

*Abbreviations*: *BMIUE* basic medical insurance for urban employees, *BMIUR* basic medical insurance for urban residents, *BMIRC* basic medical insurance for retired cadres

### Model 1: outpatient volume as the dependent variable

**Fig. 2:**
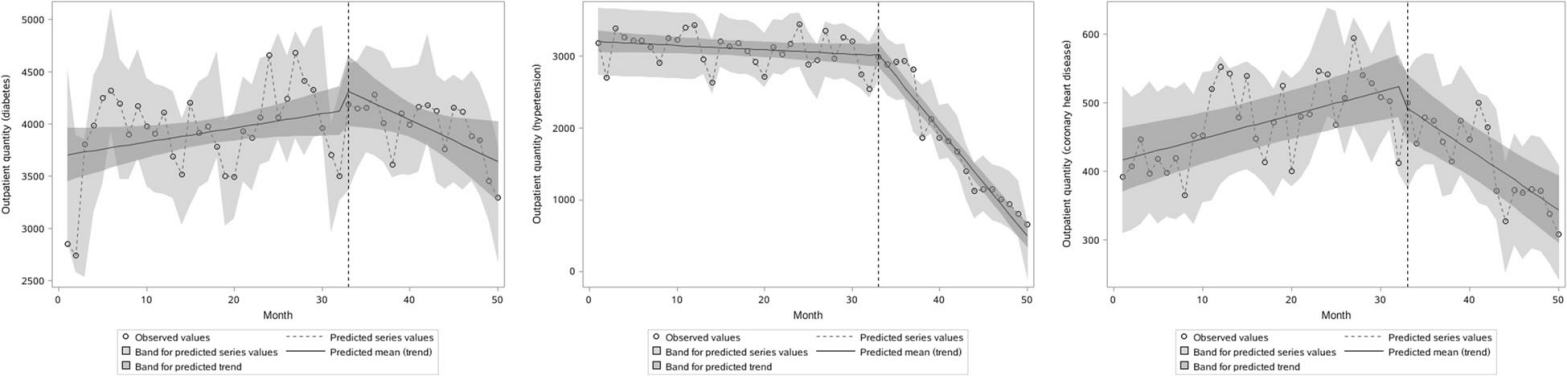
Estimated changes in outpatient volume for patients with diabetes (**a**), hypertension (**b**), and coronary heart disease (**c**)

The ITS results show that the average monthly outpatient visits for diabetes, hypertension, and CHD were 3692.00, 3211.00 and 413.38 at baseline, all of which were statistically significant (*P* < 0.001). The β1 of the three diseases was 13.63 (*P* > 0.05), − 6.05 (*P* > 0.05) and 3.45 (*P* = 0.001), shows no significant difference in the outpatient volume changes of diabetes and hypertension before ZDMP. The change of CHD outpatients’ volume was statistically significant, with an average increase of 3.45 per month. There was no significant difference in the outpatient volume after the policy in instantaneous changes (all *P* > 0.05). The three diseases’ outpatient volume has shown a downward trend after the policy. β3 of − 53.04 (*P* = 0.012), − 142.19 (*P* < 0.001) and − 12.16 (*P* < 0.001) were statistically significant. It shows that compared with before the policy, the monthly outpatient visits for diabetes, hypertension and CHD have dropped by about 53, 142, and 12, respectively (Table 2, Fig. 2). In general, after implementing ZDMP, the outpatient volume for the three diseases has shown a downward trend to varying degrees. It has the most significant impact on hypertension, followed by diabetes, and finally CHD.

**Table 2:** The parameters of the interrupted time series analysis for NCDs outpatients (outpatient volume and average drug cost as the dependent variables respectively)

Indicator		β0		β1		β2		β3	
		Estimated value	*P-*value	Estimated value	*P-*value	Estimated value	*P-*value	Estimated value	*P-*value
Outpatient volume (person-month)	Diabetes	3692.00	< 0.001	13.63	0.056	226.24	0.302	−53.04	0.012
	Hypertension	3211.00	< 0.001	−6.05	0.084	151.17	0.081	−142.19	< 0.001
	Coronary heart disease	413.38	< 0.001	3.45	0.001	−22.92	0.302	−12.16	< 0.001
Average drug cost per month (RMB)	Diabetes	761.80	< 0.001	0.16	0.835	−45.51	0.029	−4.44	0.029
	Hypertension	605.09	< 0.001	0.53	0.315	−19.79	0.276	−5.87	< 0.001
	Coronary heart disease	780.21	< 0.001	2.26	0.257	−77.46	0.047	−10.23	0.036

### Model 2: average drug cost as the dependent variable

**Fig. 3:**
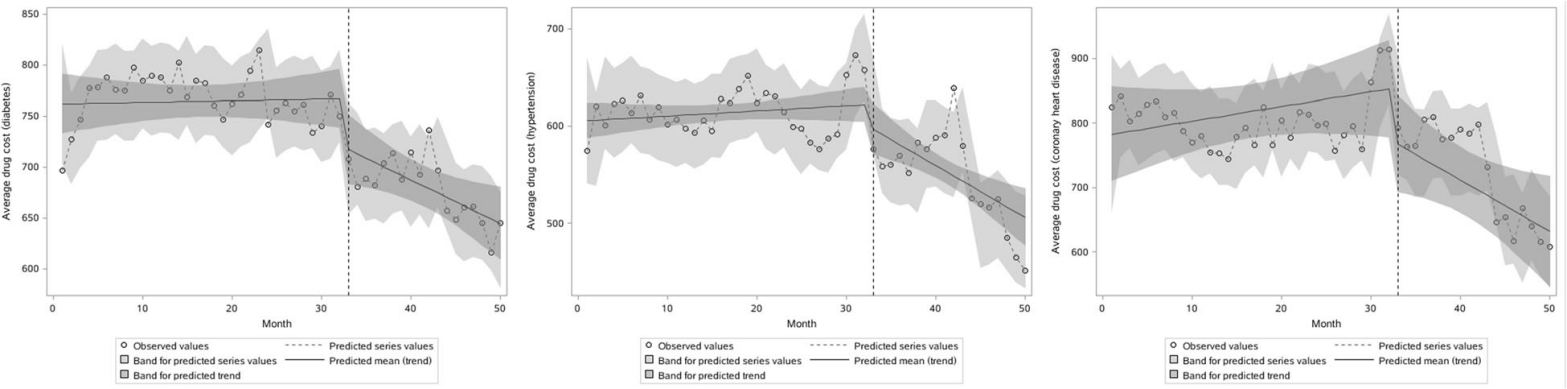
Estimated changes in average drug costs for patients with diabetes (**a**), hypertension (**b**), and coronary heart disease (**c**)

During the study period, the average drug costs for diabetes, hypertension, and CHD were 761.80, 605.09 and 780.21 yuan at baseline, all of which were statistically significant (*P* < 0.001). In terms of instantaneous changes, the β2 of these three are − 45.51, − 19.79 and − 77.46. It shows that after this policy, the average drug costs of diabetes and CHD have decreased by 45.51 yuan and 77.46 yuan, respectively, which is statistically significant (P 0.029 and 0.047, respectively). Simultaneously, there was no significant difference in transient changes in hypertension (*P* > 0.05). The average drug costs for diabetes, hypertension, and CHD have all shown a downward trend after the policy. β3 was − 4.44, − 5.87and − 10.23, all of which were statistically significant (all *P* < 0.05). It shows that compared with before the policy, the average monthly drug costs of the three have dropped by about 4.44 yuan, 5.87 yuan, and 10.23 yuan, respectively (Table 2, Fig. 3). After implementing ZDMP, the three diseases’ average drug costs have shown instantaneous changes and (or) downward trends to varying degrees. In terms of transient and trend changes (β1 + β3), it has the most significant impact on the average drug cost of CHD, followed by diabetes, and finally hypertension.

### Model 2.1: average drug cost by sex as the dependent variable

**Fig. 4:**
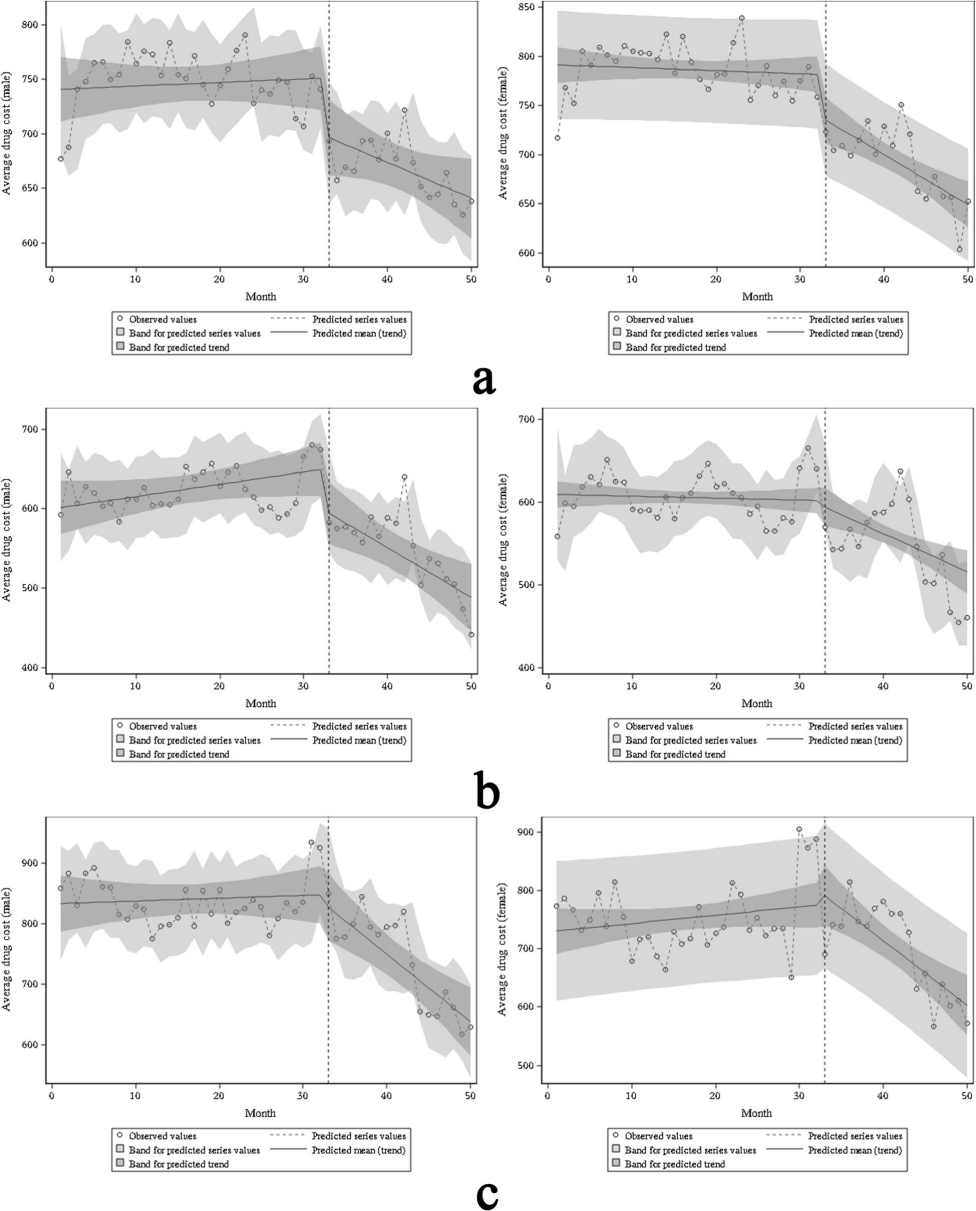
Estimated changes in average drug costs for patients of different sexes with diabetes (**a**), hypertension (**b**), and coronary heart disease (**c**). On the left is male and on the right is female

By gender comparison, the baseline average monthly drug costs of male and female patients with different diseases are statistically significant (all *P* < 0.001). Before ZDMP, there was no significant difference in all patients’ average drug costs (all *P* > 0.05). In terms of instantaneous changes, β2 in male diabetes, male hypertension and female diabetes were − 51.21 (*P* = 0.017), − 48.26 (*P* = 0.047) and − 41.24 (*P* = 0.011). After the policy, only the instantaneous changes in the above three categories’ average drug costs were statistically significant, with a decrease of 51.21 yuan, 48.26 yuan and 41.24 yuan, respectively. There was no significant difference in the instantaneous changes in men’s average drug costs with CHD, women with hypertension, and CHD (*P* > 0.05). From the perspective of trend changes, after the policy, the decline in average drug costs for male diabetic patients is not significant (β3 = − 3.62, *P* > 0.05). The downward trend of the remaining patients’ average drug cost was statistically significant (all *P* < 0.05). Specifically, male hypertension and male CHD’s long-term trend changes decreased by 7.78 yuan and 11.58 yuan per month, respectively. The long-term trends of female diabetes, hypertension, and CHD decreased by 4.78 yuan, 4.40 yuan, and 12.51 yuan (Table 3, Fig. 4).

**Table 3:** The parameters of the interrupted time series analysis for NCDs outpatients of different sexes, ages and basic medical insurance (average drug cost as the dependent variable)

Indicator		β0		β1		β2		β3	
		Estimated value	***P***-value	Estimated value	***P***-value	Estimated value	***P***-value	Estimated value	***P***-value
Sex									
Male	Diabetes	740.74	< 0.001	0.33	0.679	−51.21	0.017	−3.62	0.078
	Hypertension	600.16	< 0.001	1.54	0.097	−48.26	0.047	−7.78	0.001
	Coronary heart disease	833.00	< 0.001	0.47	0.712	−9.12	0.797	−11.58	0.001
Female	Diabetes	791.45	< 0.001	−0.30	0.547	−41.24	0.011	− 4.78	0.001
	Hypertension	610.14	< 0.001	−0.24	0.599	−2.97	0.855	−4.40	0.002
	Coronary heart disease	729.16	< 0.001	1.43	0.191	27.96	0.412	−12.51	< 0.001
Age									
0–45	Diabetes	507.08	< 0.001	1.11	0.005	−13.96	0.246	1.85	0.064
	Hypertension	353.70	< 0.001	−0.21	0.734	22.70	0.255	−3.66	0.030
	Coronary heart disease	541.37	< 0.001	3.01	0.210	−28.93	0.729	−9.22	0.163
46–65	Diabetes	720.53	< 0.001	0.33	0.336	−54.40	< 0.001	−3.16	0.001
	Hypertension	555.77	< 0.001	0.06	0.883	−20.16	0.158	−4.54	< 0.001
	Coronary heart disease	781.70	< 0.001	0.76	0.544	7.06	0.849	−11.70	< 0.001
> 65	Diabetes	874.52	< 0.001	−0.52	0.709	−55.50	0.094	−6.15	0.088
	Hypertension	689.67	< 0.001	1.16	0.306	−43.06	0.129	−8.32	0.005
	Coronary heart disease	802.53	< 0.001	2.28	0.331	−102.61	0.030	−8.91	0.117
Basic medical insurance									
BMIUE	Diabetes	800.24	< 0.001	−0.19	0.822	−53.37	0.017	−4.24	0.054
	Hypertension	623.11	< 0.001	1.02	0.170	−59.63	0.010	−5.60	0.021
	Coronary heart disease	818.94	< 0.001	1.49	0.338	−63.06	0.117	−10.02	0.010
BMIUR	Diabetes	381.93	< 0.001	1.10	0.006	−49.01	0.001	0.01	0.993
	Hypertension	281.34	< 0.001	0.69	0.349	10.71	0.682	−3.53	0.108
	Coronary heart disease	573.32	< 0.001	−0.54	0.808	−42.62	0.539	−1.27	0.822

*Abbreviations*: *BMIUE* basic medical insurance for urban employees, *BMIUR* basic medical insurance for urban residents

### Model 2.2: average drug cost by age as the dependent variable

**Fig. 5:**
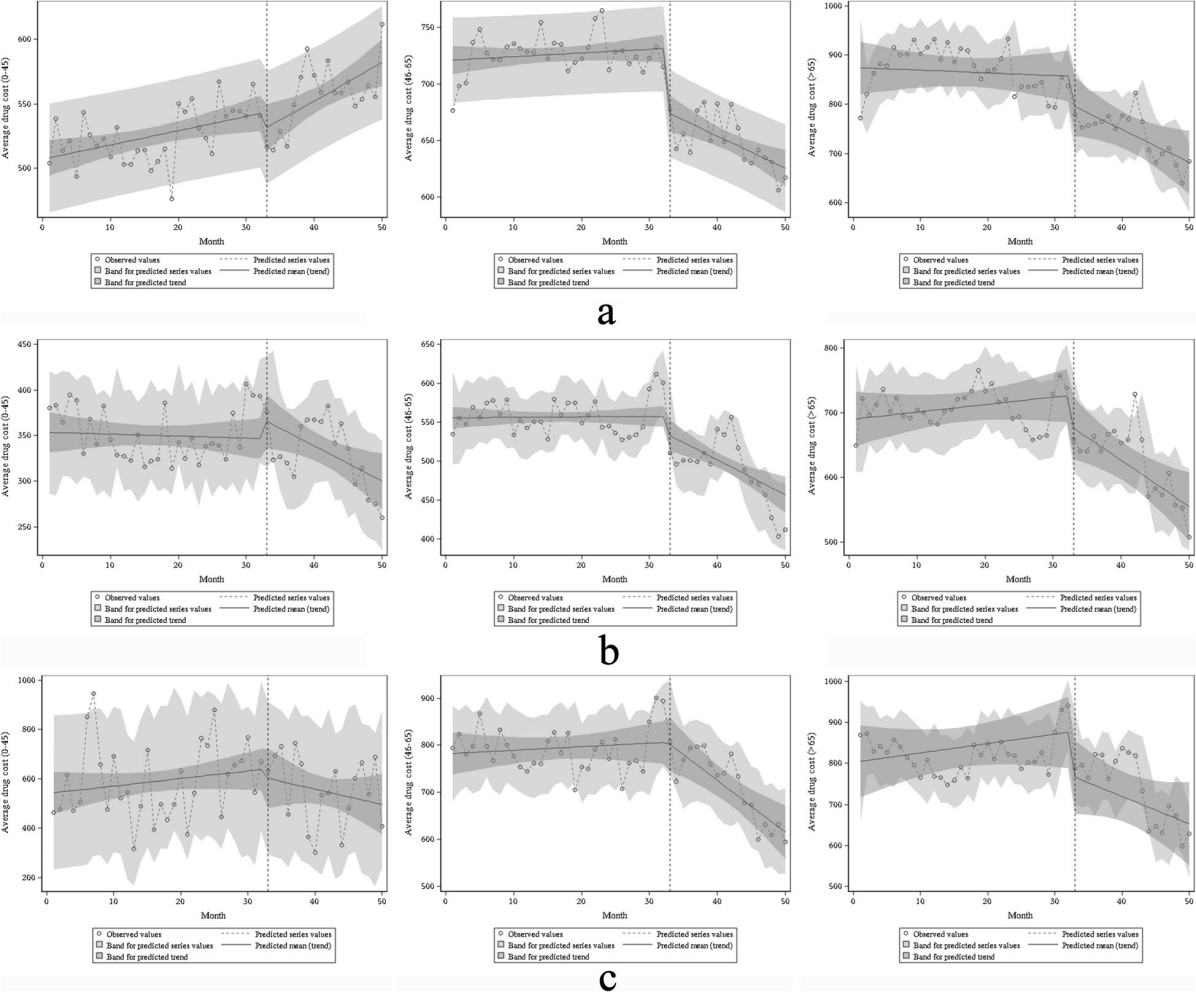
Estimated changes in average drug costs for patients of different ages with diabetes (**a**), hypertension (**b**), and coronary heart disease (**c**). On the left is the under-45 group, in the middle is the 45–65 group, and on the right is the over-65 group

A comparison of ages shows that the baseline average monthly drug costs of patients of different age groups were statistically significant (all *P* < 0.001). Before ZDMP, only the average drug cost of patients with diabetes under 46 years old had a statistically significant change (β1 was 1.11, *P* = 0.005). It shows that before the policy, the average monthly drug costs for diabetic patients < 46 years old increased by 1.11 yuan. However, there was no significant difference in the remaining patients’ average drug cost (all *P* > 0.05). In terms of instantaneous changes, β2 for diabetes with 46–65 and CHD with > 65 are − 54.4 (*P* < 0.001) and − 102.61 (*P* = 0.030), respectively. It shows that after the policy, these two patients’ average drug costs have instantaneously decreased by 54.4 yuan and 102.61 yuan. The transient changes of the remaining patients were not statistically significant (all *P* > 0.05). In terms of trend changes, the average drug costs of 46–65 years old for the three diseases have a statistically significant decline (*P* < 0.05). Specifically, the long-term trends of patients with diabetes, hypertension, and CHD decreased by 3.16 yuan, 4.54 yuan, and 11.70 yuan per month, respectively. Only the average drug cost for hypertension in the age groups of < 46 and >  65 has a significant downward trend, with a monthly decrease of 3.66 yuan and 8.32 yuan, respectively (Table 3, Fig. 5).

### Model 2.3: average drug cost by basic medical insurance type as the dependent variable

**Fig. 6:**
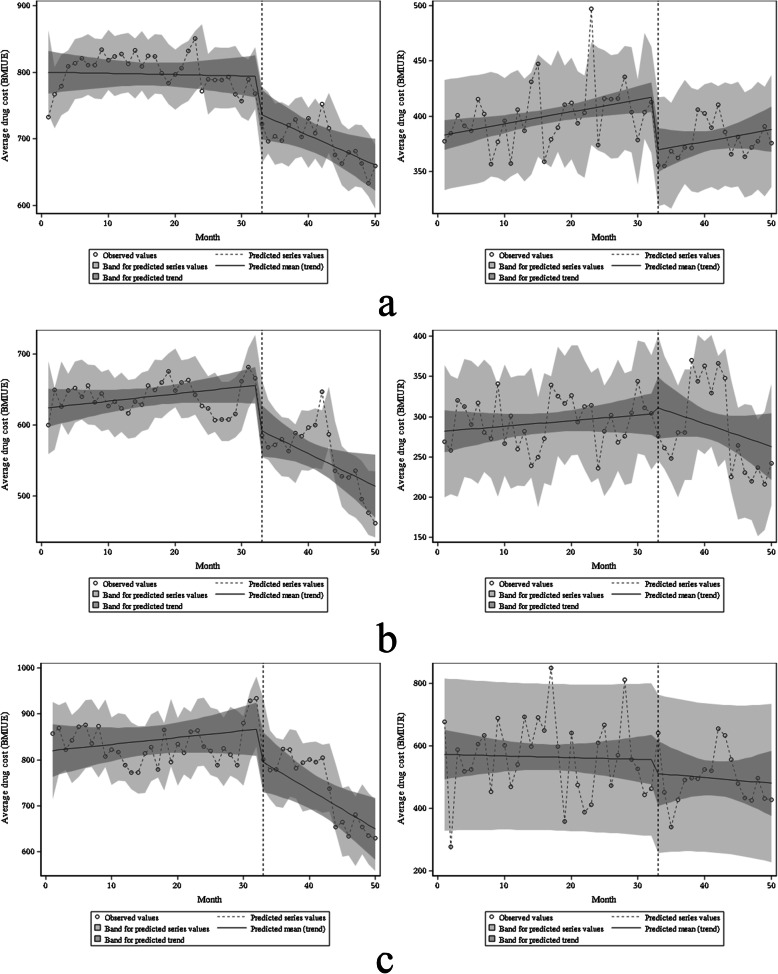
Estimated changes in average drug costs for diabetic patients (**a**), hypertensive patients (**b**), and coronary heart disease patients (**c**) under different basic medical insurance plans. On the left is BMIUE, on the right is BMIUR

The main payment methods for outpatients are BMIUE and BMIUR. The comparison found that the baseline average monthly drug costs with different medical insurance payment methods were statistically significant (*P* < 0.001). Before the ZDMP, only the average drug cost of BMIUR ‘s diabetes had a statistically significant change (β1 = 1.10, *P* = 0.006). It shows that before the policy, the average monthly drug cost of BMIUR’s diabetes increased by 1.10 yuan. There was no significant difference in the remaining patients’ average drug cost (all *P* > 0.05). In terms of transient changes, BMIUE’s diabetes, BMIUE’s hypertension, and BMIUR’s diabetes β2 were − 53.37 (*P* = 0.017), − 59.63 (*P* = 0.010) and − 49.01 (*P* = 0.001), respectively. It shows that after the policy, the average drug costs of these three types of patients have instantaneously decreased by 53.37 yuan, 59.63 yuan and 49.01 yuan, respectively. The instantaneous changes in the remaining patients’ average drug costs were not statistically significant (all *P* > 0.05). From the perspective of trend changes, the average drug costs of BMIUE’s hypertension and BMIUE’s diabetes have a statistically significant decline (*P*-value were 0.021 and 0.010, respectively), with a monthly decrease of 5.60 yuan and 10.02 yuan, respectively (Table 3, Fig. 6).

## Discussion

This study analyzes the changes in average drug costs per month for NCDs’ outpatients for 2 years after the full implementation of the ZMDP in the tertiary hospital in Chongqing. We find that after the ZMDP, the average drug costs of the three most common NCDs’ outpatients (diabetes, hypertension and coronary heart disease) all have a significant downward trend, with a monthly decrease of 4.44 yuan, 5.87 yuan and 10.23 yuan respectively (all *P* < 0.05). Zhang [29] finds that the ZMDP can reduce the prescription drug cost for hypertension in secondary hospitals by an average of 2.01 yuan, which illustrates this policy’s broad applicability. Contrary to our research, Yan found that tertiary hospitals’ average outpatient drug expenditure showed a slow downward trend before the intervention and an upward trend after the intervention [8]. It might because Yan’s research scope is too broad, and the adaptability of policies between different diseases is not considered.

We also find that different diseases have different sensitivity to the policy. The instantaneous changes in diabetes and CHD are statistically significant, and the analysis decreased by 45.51 yuan and 77.46 yuan, while there is no significant difference in CHD. This illustrates that, on the whole, the ZMDP effectively regulates the drug cost for different diseases, has universal applicability, consistent with the study’s findings by Wang [30]. Chao’s [31] research also shows that reasonable prices for essential drugs and the implementation of the ZMDP can reduce the average cost of prescriptions. But the ZMDP also puts financial pressure on hospitals. Hospitals respond to the economic impact of a sharp drop in prescription drug revenue by increasing revenue from other treatments and procedures, such as imaging tests [7]. Therefore, the evaluation of medical reform’s effect must consider the sensitivity of different diseases to policies. Simultaneously, the synergy of other indicators should also be considered comprehensively, such as total medical costs, inspection costs, and so on.

Simultaneously, the outpatient visits also experienced a downward trend. The monthly outpatient visits for diabetes, hypertension and CHD have dropped by about 53, 142, and 12, respectively, which may be synergistic with ZMDP and the hierarchical medical system [32]. “Hierarchical medical system” refers to a system by which diseases are classified according to their severity and treatment difficulty. Medical institutions of different levels undertake different diseases and gradually ensure the medical process is carried out at all levels, from general medicine to specialized therapies [33]. Implementing the hierarchical medical system has enabled patients with chronic diseases to turn to primary medical and health service institutions or nearby pharmacies for related consultations, which has reduced the number of outpatient visits in tertiary hospitals. To some extent, ZMDP plays an active role in relieving medical insurance funds’ pressure and promoting hierarchical diagnosis and treatment implementation.

The evaluation and analysis of the three diseases’ drug costs found that they are all affected by the policy. Still, our further study found that the results under different indicators are different and the results may be affected by interference factors. This study shows that the long-term trend in the average drug cost for outpatients by sex has declined after the ZMDP, indicating that the policy has practical applicability. However, further analysis revealed that ZMDP has different effects on patients of different ages with various diseases. Only patients aged 46 to 65 showed a consistent decline in the long-term trend in drug costs after the ZMDP, indicating that this policy is more beneficial to 46–65-year-old patients. This may be due to the relatively mild conditions of younger patients with treatments that require fewer drugs, so there is less change in drug costs. Our study finds that younger patients had the lowest baseline, which supports this statement, too. However, patients in the older age are often more likely to use fast-acting drugs that have higher prices due to their serious illnesses, and the demand for drugs among these patients is also greater. Therefore, the average drug cost per month has not been significantly reduced after the zero mark-up policy.

To control NCDs’ drug costs, it is also necessary to control the NCDs’ prevalence at the source and strengthen preventive investments in chronic diseases. This needs to start with primary prevention, spreading the concept of self-prevention to the public and raising awareness of chronic disease prevention among people of all ages. Among them, special attention should be paid to the compliance of elderly patients with chronic diseases, especially those with less education. Because they need to take many different drugs for a long time, a poor understanding of prescription usage and dosage may have serious consequences [34].

We find that the ZMDP has different adjustment strengths for different types of basic medical insurance. Overall, the long-term average drug cost for BMIUE patients after the ZMDP shows a continuous downward trend. For patients with BMIUR, the average drug cost for diabetic patients decreased by 49.01 yuan (*P* = 0.001). Still, there was no significant change in the average drug cost for patients with the other two diseases. In this study, the baseline level of the average drug cost for patients with BMIUE was higher than that for patients with BMIUR. This may be due to the different reimbursement ways of the two basic medical insurance plans. The BMIUR cannot reimburse outpatient expenses. However, the patients’ outpatient expenses with BMIUE can be deducted from the personal account amount, including a partial return of the paid amount. Wang’s [35] related research noted that basic medical insurance popularization had changed patients’ ability to pay and reduced patients’ sensitivity to drug costs. Besides, due to the limited reimbursement rate of patients in the BMIUR plan, such patients are more likely to choose to purchase drugs outside of the hospital independently, so the implementation of the ZMDP in tertiary hospitals has little effect on their costs. In contrast, patients enrolled in BMIUE may have a relatively higher awareness of rational drug use or are more willing to choose to purchase drugs at hospitals. Hence, the cancellation of the drug mark-up fee at public hospitals provides more significant benefits to patients enrolled in BMIUE [36]. Therefore, when the country evaluates the effects of health reforms and further reforms, it should analyze the overall situation and define specific indicators based on different medical insurance payment methods, other regions, and various medical institutions.

The contribution of this research to the literature is reflected in the following aspects. First, this research provides a useful reference on the impact of the ZMDP on the drug cost of NCDs’ outpatients. Second, in this study, a series of covariate effects were controlled to eliminate potential confounding effects. Less control over confounding factors may overestimate or underestimate specific policies’ impact. Finally, we hope this study could provide meaningful enlightenment for the subsequent deepening of the medical reform plan.

## Conclusions

Currently, research on evaluating drug costs for NCDs’ outpatients after the ZMDP lacks. This study takes Chongqing, a key city in western China, as an example and demonstrates the policy’s applicability in the field of chronic diseases by studying the impact of the ZMDP in tertiary hospitals on outpatient visits and drug costs. This study finds that the ZMDP reform can significantly reduce the medicines expenditure and the outpatient visits for different chronic diseases outpatient and regulate and control other diseases. At the same time, this study uses the ITS method to build multiple models to analyze drug costs under various confounding factors, providing a specific reference for the formulation of a deepening health reform plan. However, this study only focuses on exploring the indicator of the average drug cost. Despite the large sample size, the sample’s representativeness and the credibility of this study’s conclusions need to be improved. Further research will be carried out by increasing the evaluation index and sample size to evaluate these strategies’ long-term impact in a broader range.

## Data Availability

The data that support the findings of this study are available from the Second Affiliated Hospital of Chongqing Medical University but restrictions apply to the availability of these data, which were used under license for the current study, and so are not publicly available. Data are however available from the authors upon reasonable request and with permission of the Second Affiliated Hospital of Chongqing Medical University.

## Abbreviations

ZMDP: Zero mark-up drug policy
ITS: Interrupted time series
NCDs: Noninfectious chronic diseases
CHD: Coronary heart disease
BMIUE: Basic medical insurance for urban employees
BMIUR: Basic medical insurance for urban residents
BMIRC: Basic medical insurance for retired cadre

## Footnotes

1 The exchange rate of the RMB against the US dollar in 2019: 100 US dollars = 689.85 yuan. The data comes from the National Bureau of Statistics of China (https://data.stats.gov.cn).
